# Isolation of high-purity and high-stability exosomes from ginseng

**DOI:** 10.3389/fpls.2022.1064412

**Published:** 2023-01-12

**Authors:** Jinwoo Jang, Haewon Jeong, Eunjae Jang, Eungpil Kim, Youngdae Yoon, Sujeong Jang, Han-Seong Jeong, Geupil Jang

**Affiliations:** ^1^School of Biological Sciences and Technology, Chonnam National University, Gwangju, Republic of Korea; ^2^Biopharmaceutical Research Center, Jeonnam Bioindustry Foundation, Hwasun, Republic of Korea; ^3^Department of Environmental Health Science, Konkuk University, Seoul, Republic of Korea; ^4^Department of Physiology, Chonnam National University Medical School, Hwasun, Republic of Korea

**Keywords:** exosome, ginseng, colloidal stability, ultracentrifugation, ExoQuick system

## Abstract

Exosomes are nano-sized extracellular vesicles that regulate cell growth and defense by delivering bioactive cellular constituents. They are a promising material for biomedical and cosmetic utilization, especially in medicinal crops such as ginseng. One main hurdle to their usage is the need for a method to isolate stable exosomes with high purity. In this study, we first tested two methods to isolate exosomes from ginseng: ultracentrifugation, the most widely used method; and the ExoQuick system, a polymer-based exosome precipitation approach. We also designed and tested a third method in which we combined ultracentrifugation and ExoQuick methods. Size distribution analysis revealed that the exosome isolation purity by the ultracentrifugation and ExoQuick methods alone were 34.1% and 59.7%, respectively, while the combination method greatly improved exosome isolation purity (83.3%). Furthermore, we found that the combination method also increases the colloidal stability of isolated ginseng exosomes, and the increase was almost double that of the ultracentrifugation method. Lastly, we showed that the combination method can also be used to isolate high-purity and high-stability exosomes from the model plant *Arabidopsis*. Overall, our findings indicate that the combination method is suitable to isolate high-purity and high-stability exosomes from plants including ginseng.

## Introduction

Exosomes are nano-sized (50–150 nm) extracellular vesicles that are generated by the inward budding of endosomal membranes and secreted into the extracellular space *via* exocytosis (Tran et al., 2020; Liangsupree et al., 2021). As exosomes have a lipid bilayer structure and can transfer cellular constituents from host cells to recipient cells, it has been suggested that they play a pivotal role in cell-to-cell communication for cell growth and defense (Bobrie et al., 2011; Maia et al., 2018; Wortzel et al., 2019; Zhang et al., 2019; Vlachakis et al., 2021). Indeed, exosome contents can include bioactive substances such as cytokines, transcription factors, mRNAs, and microRNAs, and these constituents are affected by the developmental and physiological state of host cells (Gheinani et al., 2018; Pegtel and Gould, 2019; Kalluri and LeBleu, 2020). Based on these findings, exosomes have received attention as a promising resource for biomedical utilization. To date, methods to identify and use functional exosomes have been developed in animal systems. For example, exosomes isolated from human umbilical cord-derived mesenchymal stem cells (MSCs) improved fibrotic liver disease by inhibiting the epithelial-to-mesenchymal transition of hepatocytes (Li et al., 2013). In addition, exosomes isolated from human infrapatellar fat pad derived-MSCs protect articular cartilage and ameliorate gait abnormalities due to osteoarthritis *via* inhibition of the mammalian target of rapamycin (mTOR) (Wu et al., 2019).

With over 50,000 therapeutic plants in the world, medicinal crops have provided natural resources for the development of many biomedical materials (Palombo, 2011; Sasidharan et al., 2011; Katiyar et al., 2012; Ramalingum and Mahomoodally, 2014; Salmerón-Manzano et al., 2020). For instance, *Panax ginseng* is an important medicinal crop that has been widely used in East Asia, and modern studies have determined that ginseng contains a variety of bioactive constituents with therapeutic activity (Li and Li, 1973; Jayakodi et al., 2019; Cho et al., 2021). Methods to isolate biomedical compounds from ginseng for use in the biomedical and cosmetic industries have been extensively studied. Recently, ginseng-derived exosomes have attracted attention as a biomaterial. Despite the strong potential of plant exosomes for medicinal and clinical utilization, there are still challenges to overcome, such as establishing an optimized method to isolate ginseng exosomes with high purity.

In this study, we attempted to develop and optimize a method to isolate high-purity ginseng exosomes. We tested different methods including ultracentrifugation, ExoQuick, and a combined ultracentrifugation and ExoQuick method. The ultracentrifugation method includes a series of centrifugation cycles with different centrifugal forces and durations. Depending on the centrifugal force, exosomes in a suspension can be separated sequentially according to their physical properties such as the density and size of the vesicles (Stanly et al, 2016; Li et al., 2017). The ExoQuick system traps and precipitates exosomes using a polymer-based precipitation solution (Li et al., 2017; Zhang et al, 2018; Jung et al., 2020). We hypothesized that combining ultracentrifugation with the ExoQuick system would improve ginseng exosome isolation purity, and further isolation experiments using the combination method showed great improvements in exosome isolation purity. In addition, zeta potential analysis revealed that the combination method also improved the colloidal stability of ginseng exosomes. Furthermore, we successfully extracted high-purity and high-stability exosomes from the model plant *Arabidopsis* with this method, suggesting that the combination method is widely applicable for isolating high-purity and high-stability exosomes from plants including ginseng.

## Materials and methods

### Plant materials and growth conditions

The local landrace of Korean *Panax ginseng*, Jakyung, was used in this work. To isolate exosomes from ginseng, 1 g of root, stem, and leaf samples were collected from one-year-old and three-year-old ginseng plants, which were grown in an open field and purchased from a local farm at Jangseong, Korea (35°18′45.7″N, 126°45’37.8″E). For the isolation of *Arabidopsis* exosomes, *Arabidopsis thaliana* ecotype Columbia-0 (Col-0) was used. One gram of leaf samples was collected from four-week-old *Arabidopsis* plants grown in a growth chamber with a light regime of 16 h/8 h (light/dark) at 23°C.

### Exosome isolation by ultracentrifugation

Exosome isolation by ultracentrifugation was carried out as previously described with slight modification (Wang et al., 2014). Briefly, the ultracentrifugation method used in this study included two experimental steps: crude extraction by centrifugation and exosome isolation by ultracentrifugation. To obtain crude extracts from ginseng and *Arabidopsis*, 1 g of plant samples was homogenized using an HG-15D homogenizer (DAIHAN, Korea) and 10 mL of 1x phosphate-buffered saline (PBS, pH 7.4) (Biosesang, Korea). The PBS was filtered through a 0.2 μm membrane (Sartorius Stedim Biotech, Germany) before use in the exosome isolation method. Homogenized samples were centrifuged three times at 4°C to remove cell debris, fibers, and large particles (1^st^ centrifugation: 1,000 *g* for 10 mins, 2^nd^ centrifugation: 3,000 *g* for 20 mins, 3^rd^ centrifugation: 10,000 *g* for 60 mins). After the final centrifugation, supernatants were collected, and the volume was brought to 10 mL with PBS buffer. To isolate exosomes from the crude extracts, the extracts were ultra-centrifuged three times, which included a sucrose density gradient ultracentrifugation process. The first ultracentrifugation was performed at 150,000 *g* for 90 mins at 4°C using an SW 41 Ti rotor and an Optima XE-100 ultracentrifuge (Beckman Coulter, USA). The pellets formed by the first ultracentrifugation were resuspended in 1 mL of PBS buffer. Then, the resuspended samples were transferred to a sucrose gradient solution (8, 15, 30, 45, and 60% [w/v] in PBS) and centrifuged at 150,000 *g* for 90 mins at 4°C. The band between the 30% and 45% sucrose solutions was collected, which corresponds to the density of exosomes (1.12–1.20 g/cm^3^). The volume of the collected layers was brought to 10 mL with PBS buffer, and then ultra-centrifuged at 150,000 *g* for 90 mins at 4°C. The exosome pellets were resuspended in 100 μL of PBS buffer.

### Exosome purification using the ExoQuick system

Ginseng and *Arabidopsis* exosome isolation using the ExoQuick system (ExoQuick-TC™) was performed according to the manufacturer’s instructions with minor modifications (System Biosciences Inc., USA.). The ExoQuick method used in this study included two experimental steps: crude extraction by centrifugation and exosome precipitation by ExoQuick solution treatment. The crude extraction step in the ExoQuick method was identical to that in the ultracentrifugation method. To precipitate ginseng and *Arabidopsis* exosomes from crude extracts, 2 mL of the ExoQuick solution was added to 10 mL of crude extracts (crude extracts:ExoQuick solution = 5:1) and mixed well by inverting. The mixtures were incubated at 4°C overnight without rotation or mixing. The incubated mixtures were centrifuged at 1,500 g, 4°C for 30 mins. Exosome pellets were resuspended in 100 μL of PBS buffer.

### Exosome isolation using both ultracentrifugation and the ExoQuick system

The ultracentrifugation-ExoQuick combination method includes three experimental steps: a crude extraction by three rounds of centrifugation, exosome isolation by three rounds of ultracentrifugation, and exosome purification by ExoQuick solution treatment (**Supplementary Figure 1**). The crude extraction by centrifugation and exosome isolation by ultracentrifugation steps were identical to those in the ultracentrifugation method described above. In the combination method, the exosome pellets formed by the third ultracentrifugation were resuspended in 10 mL of PBS buffer and treated with 2 mL of ExoQuick solution. The subsequent precipitation step was performed identically to that in the single ExoQuick method.

### Nanoparticle tracking analysis and Zeta potential measurement

To investigate the total number and size distribution of extracellular vesicles including exosomes, Nanoparticle Tracking Analysis (NTA) was performed using Nanosight NS300™ (Malvern Instruments, UK). To do this, isolated exosome samples were diluted 1:10 with PBS buffer, and 1 mL of the diluted samples was injected into the sample chamber. NTA results were analyzed using the NTA 3.2 software (Malvern Instruments, UK). To investigate the colloidal stability of the ginseng and *Arabidopsis* exosomes isolated by the three different methods (ultracentrifugation, ExoQuick, and combination methods), zeta potential values were measured using the ELSZ-2000ZS zeta potential and particle size analyzer (Otsuka Electronics, Japan). Exosome samples were diluted 1:2 with PBS buffer, and 200 μL of samples were measured at 25°C. The results were analyzed using the ELSZ-2000 version 7.0 software (Otsuka Electronics, Japan).

### Electron microscopy observation

To visualize ginseng and *Arabidopsis* exosomes, transmission electron microscopy (TEM) was used. Isolated exosome samples were fixed in 4% paraformaldehyde for one hour, and 5 μL of fixed samples were placed on the carbon support film of a nickel grid and incubated to allow the sample to be adsorbed to the grid. Grids were washed with 60 μL of 2% uranyl acetate. TEM observation was performed using a JEM-2100F Field Emission Transmission Electron Microscope (JEOL Ltd., Japan) at 200 kV and images were captured using an UltraScan 4000 CCD camera (Gatan Inc. USA.). For cryo-transmission electron microscopy observation (Cryo-TEM), 5 μL of the isolated exosome samples were loaded onto copper Quantifoil grids with a 1.2 μm diameter hole and an inter-hole distance of 1.3 μm (Electron Microscopy Sciences, USA). Prior to freezing, the grids were glow discharged for 45 sec on each side. Grids were frozen using a Vitrobot, and the frozen samples were observed using the JEM 3200FS (JEOL Ltd., Japan) transmission electron microscope at 120 kV.

### Statistical analysis

Data are averages of at least two biological replicates with three technical replicates for each. The statistical analysis was performed using Microsoft Excel 2016, and the statistical difference between the samples and their control was determined using a two-tailed Student’s *t-*test with a *P <*0.01.

## Results

### Isolation of ginseng exosomes by ultracentrifugation

**Figure 1:**
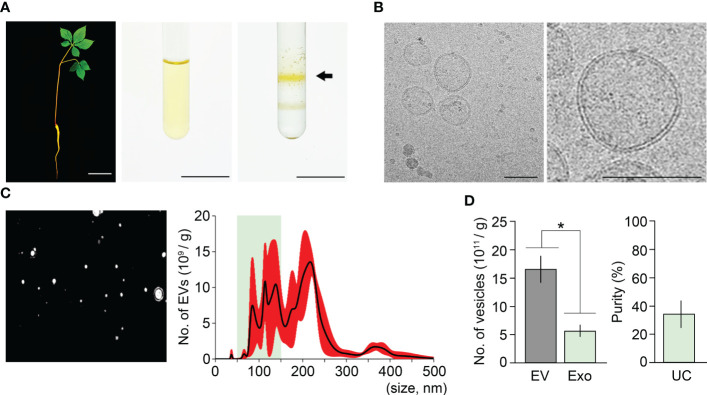
Isolation of ginseng exosomes by ultracentrifugation. **(A)** Exosome isolation from one-year-old ginseng roots (1 g) by the ultracentrifugation method (left, whole plant; middle, crude extraction by centrifugation; right, exosomal band separation by sucrose density gradient ultracentrifugation). Arrow points to the exosome-containing layer. **(B)** Visualization of isolated ginseng exosomes by cryo-transmission electron microscopy (Cryo-TEM) (left), and at higher magnification (right). **(C)** Nanoparticle Tracking Analysis (NTA) of the isolated exosome samples. Nanoparticle image (left) and size distribution results (right) were obtained using a Nanosight NS300™. The green box indicates the region containing extracellular vesicles 50–150 nm in size (exosomes), and red indicates the standard deviation (SD). **(D)** The total number of extracellular vesicles (EV) and exosomes (Exo) isolated by the ultracentrifugation method (UC) (left), and exosome isolation purity (right). Purity is expressed as a percentage (%), calculated from the ratio of the number of exosomes to the total number of isolated EVs. Error bars indicate SD. The asterisk indicates statistically significant differences between the samples (*p* value < 0.01, Student’s *t*-test). Scale bar = 3 cm in **(A)** and 100 nm in **(B)**.

Ultracentrifugation is the most widely used method to isolate exosomes (Théry et al., 2006; Li et al., 2017; Coughlan et al., 2020). To determine the purity of ginseng exosomes isolated by the ultracentrifugation method, we extracted exosomes from one-year-old ginseng roots using this method, which includes two experimental steps: crude extraction by three rounds of centrifugation, and exosome isolation by three rounds of ultracentrifugation. To obtain crude extracts, 1 g of ginseng roots collected from one-year-old ginseng plants was homogenized in 10 mL PBS buffer (**Figure 1**) and centrifuged three times at 4°C. To isolate ginseng exosomes from the crude extracts, a total of three rounds of ultracentrifugation was performed, and the second round of ultracentrifugation, which used sucrose density gradient solutions, formed two yellow layers (**Figure 1A**). The upper layer, which corresponds to the density of exosomes (1.12–1.20 g/cm^3^), was collected, and exosome pellets were obtained by the third round of ultracentrifugation. To examine whether the pellets contain exosomes, the pellets were observed by transmission electron microscopy (TEM) and Cryo-transmission electron microscopy (Cryo-TEM) (**Supplementary Figure 2**, **Figure 1B**). Although exosome-like nanoparticles were observed by TEM, this approach was not sufficient to clearly visualize whether the nanoparticles were exosomes (**Supplementary Figure 2**). To determine whether exosomes were present, we observed the samples using Cryo-TEM. The pellets obtained by the ultracentrifugation method contain ginseng exosomes, which are surrounded by a lipid bilayer and range in size between 50–150 nm, indicating that ginseng exosomes can be extracted by the ultracentrifugation method (**Figure 1B**).

To understand how the isolated EVs including exosomes are distributed in size, the samples were investigated by Nanoparticle Tracking Analysis (NTA) using Nanosight NS300™ (**Figure 1C**). The NTA approach revealed that the resuspensions contained 1.65×10^12^ EVs and that EV size ranged between 29–499 nm (**Figure 1C**). Among the EVs, the number of exosomes (50–150 nm) was around 5.62×10^11^. This suggested that ginseng exosomes make up 34.1% of the total EVs extracted by the ultracentrifugation method (**Figure 1D**).

### Isolation of ginseng exosomes using the ExoQuick system

**Figure 2:**
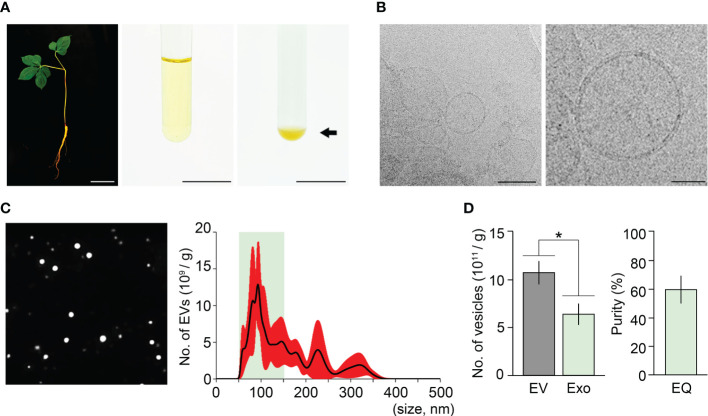
Isolation of ginseng exosomes by the ExoQuick system. **(A)** Exosome isolation from one-year-old ginseng roots (1 g) by the ExoQuick method (left, whole plant; middle, crude extraction by centrifugation; right, ginseng exosome precipitation by the ExoQuick system). Arrow points to the ginseng exosome pellet. **(B)** Visualization of the isolated ginseng exosomes by Cryo-TEM (left) and at higher magnification (right). **(C)** NTA results of the isolated exosome samples. The nanoparticle image (left) and size distribution results (right) were obtained using a Nanosight NS300™. The green box indicates the region containing extracellular vesicles 50–150 nm in size (exosomes), and red indicates SD. **(D)** Total number of extracellular vesicles (EV) and exosomes (Exo) isolated by the ExoQuick method (EQ) (left), and the exosome isolation purity (right). Purity is expressed as a percentage (%), calculated from the ratio of the number of exosomes to the total number of isolated EVs. Error bars represent SD. The asterisk indicates statistically significant differences between the samples (*p* value < 0.01, Student’s *t*-test). Scale bar = 3 cm in **(A)** and 100 nm in **(B)**.

The ExoQuick method uses a polymer-based precipitation solution, ExoQuick-TC, to isolate exosomes. ExoQuick-TC establishes a polymer network that allows exosomes to be isolated, and previous studies reported that the ExoQuick method improves exosome isolation purity in an animal cell system (Alvarez et al., 2012; Peterson et al., 2015; Jung et al., 2020). To address whether exosome isolation purity in ginseng can be improved by the ExoQuick system, we attempted to extract exosomes from one-year-old ginseng roots using this system. The ExoQuick method we designed in this approach included two experimental steps; crude extraction by three rounds of centrifugation, and exosome precipitation by ExoQuick solution treatment. The crude extraction step in the ExoQuick method was identical to that in the ultracentrifugation method. Crude extracts obtained from 1 g of one-year-old ginseng roots were treated with ExoQuick solution to precipitate exosomes (crude extract:ExoQuick solution = 5:1), and the pellets were resuspended in 100 μL PBS buffer (pH 7.4) (**Figure 2A**). When the resuspended samples were visually examined using Cryo-TEM, exosomes surrounded by a lipid bilayer were observed (**Figure 2B**). These findings suggested that EVs, including exosomes, can be isolated by the ExoQuick method.

To investigate the size distribution of isolated EVs, NTA was performed. The NTA result showed that the resuspended solutions contained 1.08×10^12^ EVs, and their size ranged between 42–389 nm (**Figure 2C**). Among the total EVs isolated by the ExoQuick method, the number of exosomes (50–150 nm) was approximately 6.4×10^11^. This finding indicated that ginseng exosomes make up 59.7% of the total EVs extracted by the ExoQuick system, suggesting that the ExoQuick method extracts exosomes from ginseng roots with 59.7% purity (**Figure 2D**). The exosome isolation purity of the ExoQuick method was approximately 75% higher than that obtained using the ultracentrifugation method (34.1%). These data indicate that exosome isolation purity is affected by isolation methods and that the ExoQuick system improves exosome isolation purity in ginseng. Also, this finding suggested that combining ultracentrifugation and the ExoQuick system may further improve the isolation purity of ginseng exosomes.

### Isolation of ginseng exosomes by combining ultracentrifugation and the ExoQuick system

**Figure 3:**
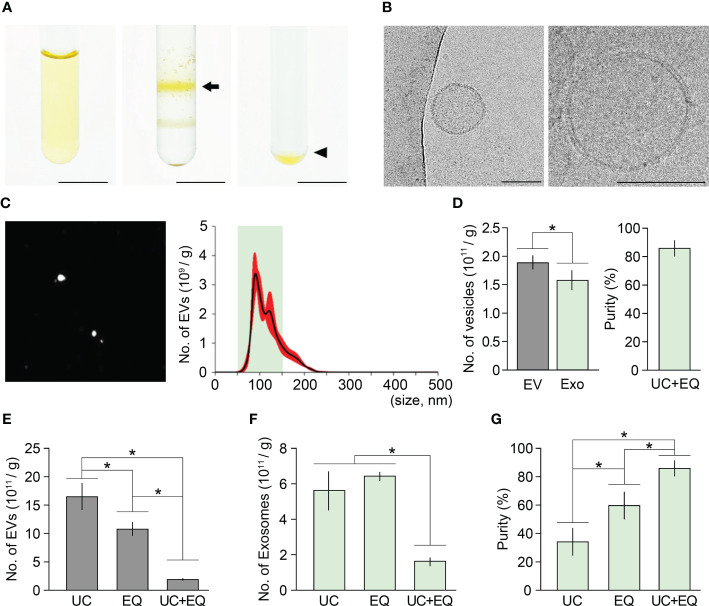
Improvement of ginseng exosome isolation purity by the combination of ultracentrifugation and ExoQuick system. **(A)** Exosome isolation from one-year-old ginseng roots (1 g) by combining ultracentrifugation (UC) and the ExoQuick (EQ) system (left, crude extraction by centrifugation; middle, exosomal band separation by sucrose density gradient ultracentrifugation; right, exosome precipitation by the ExoQuick system). The arrow and arrowhead indicate the exosome-containing layer and the exosome pellet, respectively. **(B)** Cryo-TEM image of exosomes isolated by the combination method (left) and visualized at higher magnification (right). **(C)** NTA results of the isolated exosome samples. Nanoparticle image (left) and size distribution results (right) were obtained using a Nanosight NS300™. The green box indicates the region containing extracellular vesicles 50–150 nm in size (exosomes), and red indicates SD. **(D)** Total number of extracellular vesicles (EV) and exosomes (Exo) isolated by the combination of ultracentrifugation and ExoQuick method (UC+EQ) (left), and exosome isolation purity (right). Purity is expressed as a percentage (%), calculated from the ratio of the number of exosomes to the total number of isolated EVs. **(E-G)** Comparative analysis of exosome samples isolated by ultracentrifugation alone (UC), ExoQuick alone (EQ), and the combination of ultracentrifugation and ExoQuick (UC+EQ). **(E)** number of EVs; **(F)** number of exosomes; **(G)** exosome isolation purity). Error bars represent SD and asterisks indicate statistically significant differences between the samples (*p* value < 0.01, Student’s *t*-test). Scale bar = 3 cm in **(A)** and 100 nm in **(B)**.

To test the hypothesis that exosome isolation purity in ginseng can be improved by applying a combination of ultracentrifugation and the ExoQuick method, we designed a combination method that includes three experimental steps: crude extraction by centrifugation, exosome isolation by ultracentrifugation, and exosome precipitation by ExoQuick-TC treatment. We then attempted to extract ginseng exosomes using this combination method (**Figure 3A**). Sucrose gradient ultracentrifugation of the crude extracts formed two yellow layers and the upper layer was collected and precipitated using ExoQuick-TC™ solution to obtain exosome pellets. When the pellets were observed using Cryo-TEM, 50–150 nm vesicles with membrane structure were detected (**Figure 3B**), indicating that the pellets included ginseng exosomes. To address how the combination method affects exosome isolation purity, we investigated the total number of EVs and their size distribution by an NTA assay. The total number of EVs in the samples was 1.87×10^11^, and EV sizes ranged between 43–244 nm (**Figures 3C, D**). Among the total EVs, the number of exosomes was 1.56×10^11^, indicating that approximately 83% of the total isolated EVs were exosomes. This result suggested that combining ultracentrifugation and the ExoQuick system significantly improves exosome isolation purity in ginseng. Exosome isolation purity using the combination method was approximately 2.4-fold and 1.5-fold higher than that of the ultracentrifugation and ExoQuick methods alone, respectively (**Figures 3E–G**; **Supplementary Table 1**).

### Combination method is a suitable tool for isolating high-purity exosomes in plants

**Figure 4:**
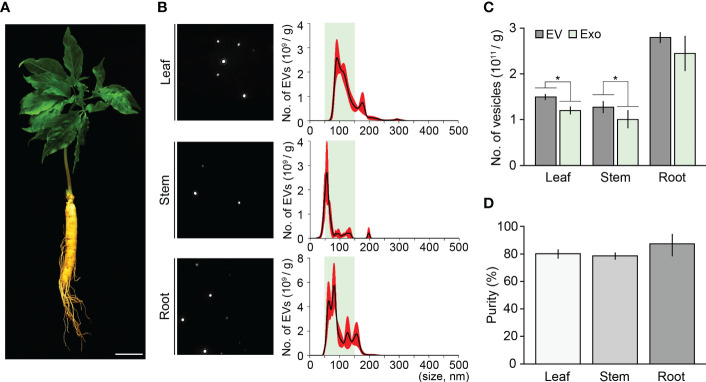
Validating the combination method using various ginseng tissues. **(A)** Image of a three-year-old ginseng plant. **(B)** NTA results of ginseng exosome sample extracted from 1 g of leaves, stems, and roots by the combination method. Green boxes represent regions containing extracellular vesicles 50–150 nm in size (exosomes), and red indicates SD. **(C, D)** Total number of isolated extracellular vesicles (EV) and exosomes (Exo) **(C)**, and exosome isolation purity **(D)**. Purity is expressed as a percentage (%), calculated from the ratio of the number of exosomes to the total number of isolated EVs. Error bars represent SD. Asterisks indicate statistically significant differences between the samples (*p* value < 0.01, Student’s *t*-test). Scale bar = 3 cm.

**Figure 5:**
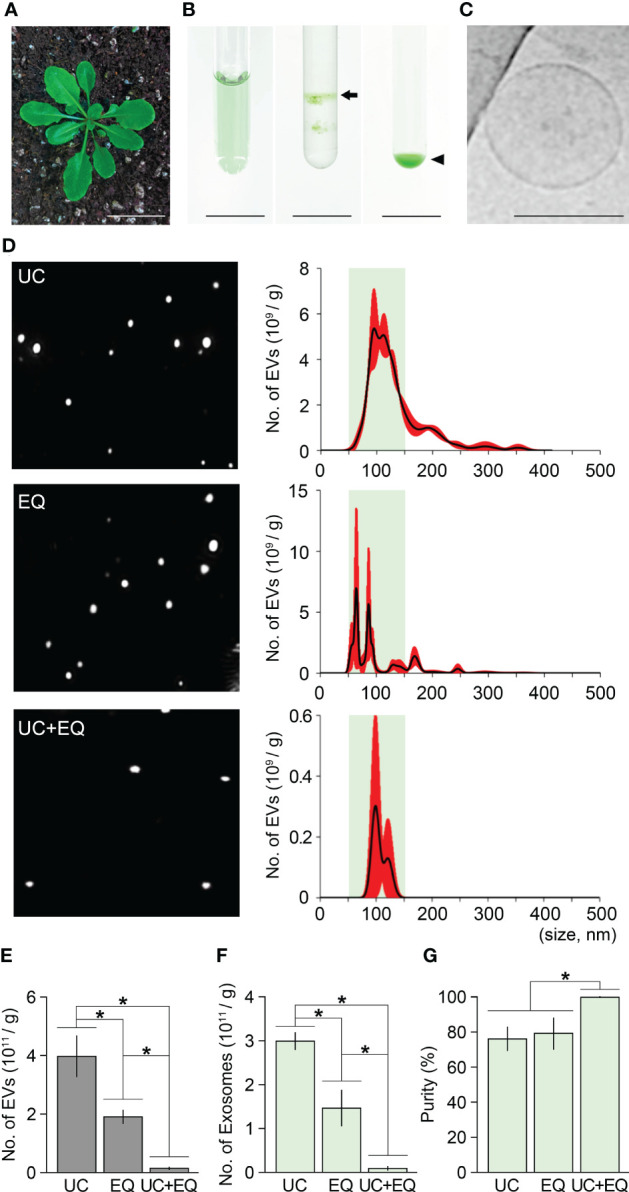
Isolation of *Arabidopsis* exosomes by the combination method. **(A)** A four-week-old *Arabidopsis* plant. **(B)** Exosome isolation from *Arabidopsis* leaves (1 g) by the combination method (left, crude extraction by centrifugation; middle, exosomal band separation by sucrose density gradient ultracentrifugation; right, exosome precipitation by the ExoQuick system). Arrow and arrowhead indicate the exosome-containing layer and exosome pellet, respectively. **(C)** Cryo-TEM image of *Arabidopsis* exosomes isolated by the combination method. **(D)** NTA results of ginseng exosome samples extracted by ultracentrifugation alone (UC), ExoQuick alone (EQ), and the combination method (UC+EQ). Green boxes represent regions containing extracellular vesicles 50–150 nm in size (exosomes), and red indicates the SD. **(E-G)** Comparative analysis of *Arabidopsis* exosome samples isolated by the three different methods (E, number of extracellular vesicles, EVs; F, number of exosomes; G, exosome isolation purity). Error bars represent SD and asterisks indicate statistically significant differences between samples (*p* value < 0.01, Student’s *t*-test). Scale bars = 3 cm in **(A)**, 100 nm in **(B)**.

To address whether the ultracentrifugation-ExoQuick combination method can extract high-purity exosomes from different tissues or different developmental stages of ginseng, we applied this method to leaves, stems, and roots of three-year-old ginseng plants, and then performed an NTA assay (**Figures 4A, B**). The total number of EVs isolated from 1 g of leaves, stems, and roots were 1.49×10^12^, 1.27×10^12^, and 2.79×10^12^, respectively. Although EVs smaller than 50 nm or bigger than 150 nm were partially detected, most EVs in the extracts were between 50–150 nm. In the leaf, stem, and root extracts, the number of exosomes was 1.20×10^12^, 1.00×1012 and 2.44×10^12^, respectively, indicating that exosome isolation purity from these tissues was 80.1%, 78.9%, and 87.4%, respectively (**Figures 4C, D**). Furthermore, the exosome isolation purity of the three-old-year ginseng plants was almost identical to that of the one-year-old ginseng plants (**Supplementary Figure 3**). These findings suggested that the ultracentrifugation-ExoQuick combination method is a suitable technique to extract exosomes with high purity in plants including ginseng, and this finding was supported by an exosome isolation test using the model plant *Arabidopsis* (**Figure 5**).

To obtain *Arabidopsis* crude extracts, 1 g of leaves from four-week-old plants was homogenized using 10 mL PBS buffer (pH 7.4) and centrifuged three times (**Figure 5A**). Sucrose gradient ultracentrifugation of the crude extracts formed two green layers, and the upper layer was treated with ExoQuick-TC solution to precipitate exosomes (**Figure 5B**). As expected, Cryo-TEM visualization revealed that the *Arabidopsis* pellets contained membrane-bound vesicles 50–150 nm in size, suggesting that *Arabidopsis* exosomes can also be extracted by the combination method (**Figure 5C**). In addition, by comparing the NTA results of EV samples extracted by ultracentrifugation alone, ExoQuick alone, and the ultracentrifugation-ExoQuick combination method, it was found that the combined method improves exosome isolation purity even in *Arabidopsis* (**Figures 5D–G**). In the *Arabidopsis* EV samples extracted by the ultracentrifugation and ExoQuick methods alone, exosome purity was 75.9% and 78.7% respectively. In contrast, the combination method increased exosome purity to approximately 100% (**Figure 5G**). These findings indicated that the combination method increases exosome isolation purity by around 23% compared with that achieved by ultracentrifugation and ExoQuick methods alone in *Arabidopsis*. The data also suggest that the combination method can be widely used for the isolation of high-purity exosomes from plants including ginseng.

### Combination method also improves colloidal stability of isolated exosomes

**Figure 6:**
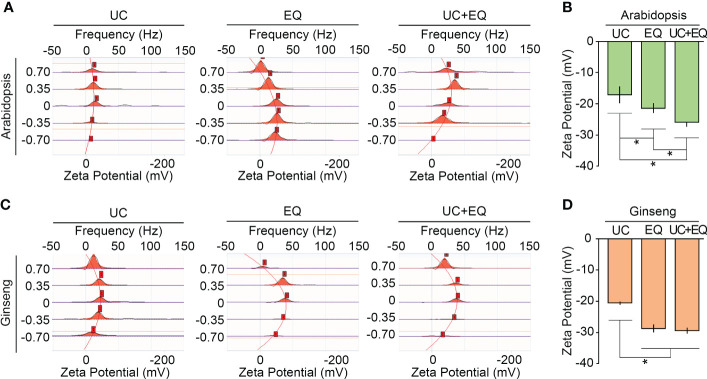
The combination method improves the colloidal stability of isolated exosomes. Zeta potential measurements of *Arabidopsis* **(A, B)** and ginseng **(C, D)** exosomes isolated by ultracentrifugation (UC), ExoQuick (EQ), and the combination (UC+EQ) methods. For this analysis, exosomes were isolated from 1 g of four-week-old *Arabidopsis* leaves and one-year-old ginseng roots. Error bars represent SD and asterisks indicate statistically significant differences between the samples (*p* value < 0.01, Student’s *t*-test).

Colloidal stability is a key factor determining the functional maintenance and storage of isolated exosomes. The colloidal stability of isolated exosomes is greatly affected by isolation methods and sample types (Jeyaram and Jay, 2018; Midekessa et al., 2020). Zeta potential analysis, which measures the surface potential of colloidal particles, is widely used to estimate the colloidal stability of isolated exosomes. Previous studies suggested that the zeta potentials of isolated exosomes range between −6 and −30 mV, and values less than −20 mV indicate high colloidal stability (Lee, 1998; Soares Martins et al., 2018; Kim et al., 2021). The zeta potentials of *Arabidopsis* exosomes extracted by the three different methods were measured and compared. As expected, the zeta potentials were significantly different among the isolation methods, and we found that the ExoQuick system decreased zeta potentials (**Figures 6A, B**; **Supplementary Table 2**). The zeta potential of exosomes isolated by the ultracentrifugation method was −17.1 mV. Exosomes isolated by the ExoQuick method alone and by the combined ultracentrifugation-ExoQuick method had zeta potential values of −21.3 and −25.9 mV, respectively. This suggested that the ExoQuick system increased the colloidal stability of isolated exosomes, and that combining ultracentrifugation with the ExoQuick system is most effective to improve the colloidal stability of isolated *Arabidopsis* exosomes. This finding was further supported by the zeta potentials of ginseng exosomes. The zeta potential of ginseng exosomes extracted by the ultracentrifugation method was −20.61 mV. The zeta potentials of exosomes isolated by the ExoQuick and the combination methods were −28.88 and −29.54 mV, respectively (**Figures 6C, D** ). Collectively, these findings suggest that combining ultracentrifugation with the ExoQuick system is an effective method to isolate high-purity and high-stability exosomes from plants including ginseng.

## Discussion

Exosomes carry a variety of bioactive compounds that regulate cell growth and defense (Akuma et al., 2019; Xia et al., 2019; Fu et al., 2020; Pinedo et al., 2021; Song et al., 2021). There is increasing evidence that plant-derived exosomes can have therapeutic effects and health benefits (Ju et al., 2013; Mu et al., 2014; Raimondo et al., 2015; Zhuang et al., 2015; Deng et al., 2017; Munir et al., 2020; Cho et al., 2021; Kim et al., 2021; Cai et al., 2022). For example, exosomes or exosome-like nanoparticles isolated from grapes strongly promote the division of intestinal stem cells and regeneration of mucosal epithelium in the dextran sulfate sodium-induced colitis mouse model (Ju et al., 2013). Similarly, broccoli-derived nanovesicles have preventive and therapeutic effects for colitis (Deng et al., 2017). In addition, exosomes or exosome-like nanovesicles isolated from lemon and ginger have anti-cancer activity and protective effects against alcohol-induced liver damage, respectively (Raimondo et al., 2015; Zhuang et al., 2015; Cho et al., 2021). This strongly suggests the potential application of plant exosomes in the pharmaceutical and cosmetic industries and highlights the importance of an optimized method to isolate plant exosomes with high purity and stability. In this study, we determined that combining ultracentrifugation with the ExoQuick system allows for the extraction of plant exosomes with higher purity than the ultracentrifugation or ExoQuick methods alone.

In *Arabidopsis*, the purity of exosomes isolated by ultracentrifugation or the ExoQuick method was approximately 70%. However, the purity of exosomes isolated by the combination method was almost 100%, indicating that this method increases isolation purity by 30% in *Arabidopsis* compared with ultracentrifugation or ExoQuick methods alone. The increase in isolation purity of the combination method was more dramatic in ginseng. The purity of exosomes isolated by ultracentrifugation or by the ExoQuick method was 34% and 59%, respectively, and that of the combination method was 83%. This indicates that the combination method increases ginseng exosome isolation purity approximately 2.4-fold and 1.5-fold compared with the single methods. These results suggest that the combination of ultracentrifugation and the ExoQuick system is an effective and stable method to extract ginseng exosomes with high purity, which is further supported by our data showing that the combination method can also be applied to various ginseng tissues. However, as expected, the increase in exosome isolation purity was accompanied by a decrease in the total number of isolated exosomes. For example, the total number of ginseng exosomes isolated *via* the ultracentrifugation, ExoQuick, and combination methods were 5.62×10^11^, 6.45×10^11^, and 1.56×10^11^ respectively. Therefore, the combination method decreases the total number of exosomes by approximately four-fold compared with the single methods. These findings indicate that while the combination method improves isolation purity, there is a reduction in the total number of exosomes obtained.

Colloidal stability is an important factor influencing the functional maintenance and storage stability of isolated exosomes. Flocculation of exosomes negatively affects their colloidal stability (Mu et al., 2014; Wang et al., 2015; Helwa et al., 2017; Umar et al., 2018; Kim et al., 2022). Zeta potential is key to estimating the colloidal stability of isolated exosomes (Clogston and Patri, 2011; Soares Martins et al., 2018; Samimi et al., 2019; Midekessa et al., 2020). In this study, we showed that the ultracentrifugation-ExoQuick combination method improved the colloidal stability of exosomes. In ginseng, the zeta potential of exosomes extracted by the combination method was −29.54 mV, which was 43% lower than that of exosomes extracted by the ultracentrifugation method. Similarly, in *Arabidopsis*, the zeta potential of exosomes isolated by the combination method was −25.89 mV, which was approximately 50% lower than that of exosomes isolated by the ultracentrifugation method. These data suggest that the combination method improves the colloidal stability of isolated exosomes as well as the isolation purity.

Our NTA results suggested that the improvement of colloidal stability is not directly caused by a decrease in isolated exosome numbers. For example, the total number of ginseng exosomes isolated by ultracentrifugation was around two-fold higher than that of *Arabidopsis* exosomes isolated by the same method, but the zeta potentials of ginseng exosomes were approximately 20% lower than that of *Arabidopsis* exosomes. In addition, the total number of ginseng exosomes isolated by the combination method was four-fold lower than that of the ExoQuick method alone, but their zeta potential values were similar. Instead, we noticed that all exosome samples isolated by methods including the ExoQuick precipitation step displayed lower zeta potential values than those isolated by the ultracentrifugation method without the ExoQuick precipitation step. This finding suggests that the ExoQuick precipitation step is likely key to improving the colloidal stability of isolated exosomes. Collectively, our findings indicate that the ultracentrifugation-ExoQuick combination method improves both the isolation purity and colloidal stability of ginseng exosomes. We propose that this technology may be widely used in scientific research as well as for industrial applications of ginseng exosomes.

## Data availability statement

The original contributions presented in the study are included in the article/**Supplementary Material**. Further inquiries can be directed to the corresponding author.

## Author contributions

GJ conceived the original screening and research plans. SJ, HJ, and GJ designed and supervised the experiments. JJ, HJ, EK, and EJ performed the experiments and analyzed the data. YY and GJ wrote the article with contributions from all authors. GJ agrees to serve as the author responsible for contact and ensures communication. All authors contributed to the article and approved the submitted version.
