# Supplementary material for: Isolation of high-purity and high-stability exosomes from ginseng
Source: Front Plant Sci. 2023 Jan 12;13:1064412. doi: 10.3389/fpls.2022.1064412 (PMC9878552; doi:10.3389/fpls.2022.1064412)
Supplement: Supplementary file 1 [file DataSheet_1.pdf]

Supplementary Material

Crude extraction

1. Sample homogenization using 10 mL of PBS buffer (pH 7.4)
2. First centrifugation of homogenized sample : 1,000 *g* for 10 mins at 4°C
3. Second centrifugation : 3,000 *g* for 20 mins at 4°C
4. Third centrifugation : 10,000 *g* for 60 mins at 4°C
5. Volume brought up to 10 mL with PBS buffer

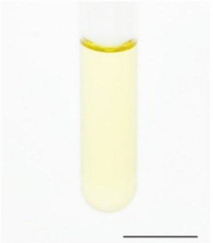

Exosome isolation by ultracentrifugation

6. First ultracentrifugation of the crude extracts : 150,000 *g* for 90 mins at 4°C
7. Pellet resuspension in 1 mL of PBS buffer
8. Sucrose density gradient ultracentrifugation : 150,000 *g* for 90 mins at 4°C
9. Collection of the exosome layers and volume brought up to 10 mL with PBS buffer
10. Third ultracentrifugation of the collected layers : 150,000 *g* for 90 mins at 4°C
11. Pellet resuspension in10 mL of PBS buffer

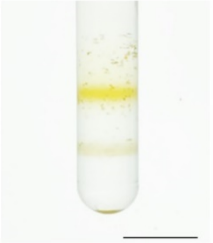

Exosome purification by ExoQuick

12. ExoQuick solution (2 mL) added to the 10 mL resuspended pellet sample
13. Incubation at 4°C overnight without rotation or mixing
14. Centrifugation at 1,500 *g* for 30 mins at 4°C
15. Pellet resuspension in 100 μL of PBS buffer

Supplementary Figure 1. The experimental procedure of the combination method. The combination method includes three experimental steps: crude extraction, exosome isolation by ultracentrifugation, and exosome purification by ExoQuick. Briefly, the crude extraction was performed by three rounds of centrifugation, and exosome isolation was performed by three rounds of ultracentrifugation including sucrose density gradient ultracentrifugation. Exosome purification was carried out by treatment with ExoQuick solution and centrifugation. Details are described in the Materials and methods section. Scale bars = 3 cm.

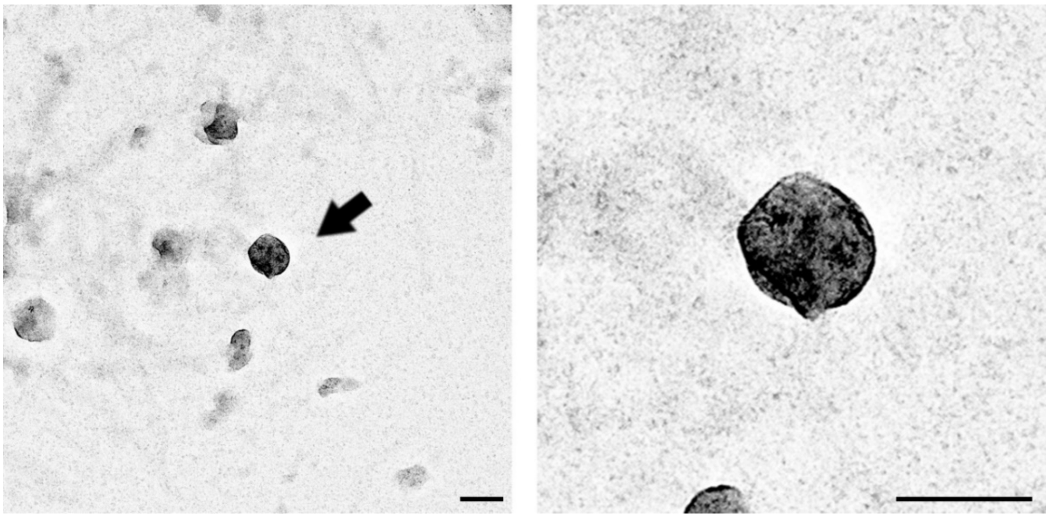

Supplementary Figure 2. Visualization of isolated ginseng exosomes by transmission electron microscopy. Transmission electron microscope (TEM) image of ginseng exosomes (left), and exosomes shown at a higher magnification (right). Ginseng exosomes were isolated from one-year-old ginseng roots using the ultracentrifugation method. Scale bars = 100 nm.

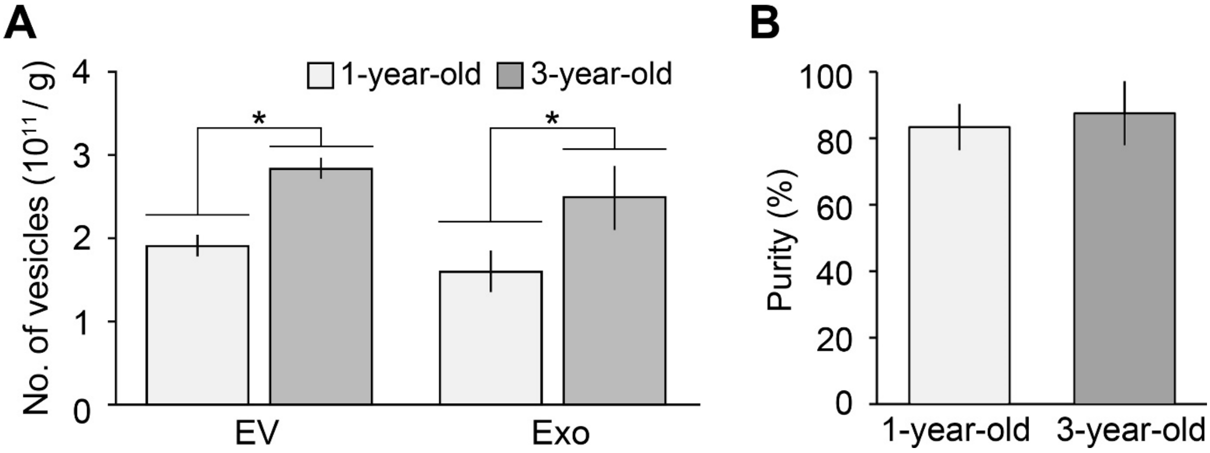

Supplementary Figure 3. Purity of ginseng exosomes isolated by the combination method. The total number of extracellular vesicles (EV) and exosomes (Exo) isolated from 1 g of roots collected from one-year-old and three-year-old ginseng plants by the combination method **(A)**, and the corresponding exosome isolation purity **(B)**. Purity is expressed as a percentage, calculated from the ratio of the number of exosomes to the total number of isolated EVs. Error bars represent SD. Asterisks indicate statistically significant differences between samples (p value < 0.01, Student's t-test).

Supplementary Table 1. Purity of ginseng and Arabidopsis exosomes isolated by three different methods.

Plant	Isolation method	No. of EVs (particle / g)	No. of exosomes (particle / g)	Purity (%)
Ginseng	Ultracentrifugation	$1.65 \times 10^{12} \pm 2.39 \times 10^{11}$ **a**	$5.62 \times 10^{11} \pm 1.11 \times 10^{11}$ **a**	34.11 ± 9.70 **a**
	ExoQuick	$1.08 \times 10^{12} \pm 1.22 \times 10^{11}$ **b**	$6.45 \times 10^{11} \pm 2.97 \times 10^{10}$ **a**	59.75 ± 10.80 **b**
	Ultracentrifugation + ExoQuick	$1.87 \times 10^{11} \pm 1.23 \times 10^{10}$ **c**	$1.56 \times 10^{11} \pm 2.48 \times 10^{10}$ **b**	83.33 ± 5.74 **c**
Arabidopsis	Ultracentrifugation	$3.96 \times 10^{11} \pm 7.14 \times 10^{9}$ **a**	$3.00 \times 10^{11} \pm 1.96 \times 10^{11}$ **a**	75.93 ± 7.01 **a**
	ExoQuick	$1.87 \times 10^{11} \pm 2.33 \times 10^{10}$ **b**	$1.47 \times 10^{11} \pm 4.14 \times 10^{10}$ **a**	78.78 ± 9.16 **a**
	Ultracentrifugation + ExoQuick	$9.23 \times 10^{9} \pm 3.66 \times 10^{9}$ **c**	$9.23 \times 10^{9} \pm 3.66 \times 10^{9}$ **b**	99.99 ± 0.03 **b**

Different letters indicate statistically significant differences (p value < 0.01, Student's t-test) in the number of extracellular vesicles, the number of exosomes, and exosome purity among the different isolation methods for each plant type.

Supplementary Table 2. Colloidal stability of ginseng and Arabidopsis exosomes.

Plant	Isolation method	Zeta Potential (mV)
Arabidopsis	Ultracentrifugation	-17.09 ± 2.74 [a]
	ExoQuick	-21.32 ± 1.57 [b]
	Ultracentrifugation + ExoQuick	-25.89 ± 1.47 [c]
Ginseng	Ultracentrifugation	-20.61 ± 0.50 [a]
	ExoQuick	-28.88 ± 1.42 [a]
	Ultracentrifugation + ExoQuick	-29.54 ± 0.98 [b]

Different letters indicate statistically significant differences (p value < 0.01, Student's t-test) in zeta potentials among isolation methods for each plant type.
